# Supplementary material for: Longitudinal transmission of bacterial and fungal communities from seed to seed in rice
Source: Commun Biol. 2022 Aug 1;5:772. doi: 10.1038/s42003-022-03726-w (PMC9343636; doi:10.1038/s42003-022-03726-w)
Supplement: Supplementary file 2 — Supplementary Information [file 42003_2022_3726_MOESM2_ESM.pdf]

Supplementary Information for

# Longitudinal transmission of bacterial and fungal communities from seed to seed in rice

**Authors**

Hyun Kim[1], Jongbum Jeon[2]†, Kiseok Kieth Lee[1]‡, and Yong-Hwan Lee[1,2,3,4,5]*

**Affiliations**

[1]Department of Agricultural Biotechnology, Seoul National University, Seoul 08826, Republic of Korea

[2]Interdisciplinary Program in Agricultural Genomics, Seoul National University, Seoul 08826, Republic of Korea

[3]Center for Plant Microbiome Research, Seoul National University, Seoul 08826, Republic of Korea

[4]Plant Immunity Research Center, Seoul National University, Seoul 08826, Republic of Korea

[5]Research Institute of Agriculture and Life Sciences, Seoul National University, Seoul 08826, Republic of Korea

†Present address: Korea Bioinformation Center, Korea Research Institute of Bioscience and Biotechnology, Daejeon 34141, Republic of Korea

‡Present address: Department of Ecology and Evolution, The University of Chicago, 1101 East 57th Street, Chicago, Illinois, 60637 USA

*Correspondence:

Yong-Hwan Lee

E-mail) yonglee@snu.ac.kr

**This PDF file includes:**

Supplementary Results 1 to 3

Supplementary Figs. 1 to 12

Supplementary Tables 1 to 2

## Supplementary Result 1

### Vertically transmitted OTUs compose core microbial communities of aboveground compartments

To find whether vertically transmitted OTUs are systemically distributed in stem endosphere along with rice development, we compared the profiles of prevalence-based core OTUs and vertically transmitted OTUs in rice compartments including stem endosphere. Core OTUs were defined as OTUs detected at 90% of prevalence across different developmental stages in each compartment. Based on this definition, core bacterial and fungal OTUs were revealed from each compartment (seed, leaf, stem, root, rhizosphere, and bulk soil; Supplementary Data 5). In the bacterial community, 17 out of 29 transmitted OTUs were identified as core OTUs of at least one aboveground compartment (Fig. 4c). Similar to the bacterial OTUs, 21 out of 34 fungal transmitted OTUs were identified as core OTUs of at least one aboveground compartment (Fig. 4c). Among 17 bacterial and 21 fungal transmitted OTUs, 11 bacterial and 17 fungal OTUs were found as core OTUs of stem endosphere. These results indicate that vertically transmitted OTUs can colonize the stem endosphere during lifetime of rice. The presence pattern of vertically transmitted OTUs in stem sections (stems divided at 10 cm interval) further demonstrated that vertically transmitted OTUs can move from bottom to the top of stem endosphere following the elongation of rice stem (Supplementary Fig. 9). Apart from stem endosphere, vertically transmitted OTUs also could colonize in the endosphere of leaves regardless of their relative abundances (Fig. 4d). Vertically transmitted but non-core OTUs were frequently distributed in the leaf and stem endosphere in the life cycle of rice.

**Supplementary Result 2**

**Similarity of microbial associations during rice development**

To find the similarity of microbial associations among rice compartments during the development, we compared edge profiles of 101 interkingdom co-occurrence networks using a Jaccard similarity index (*J*). Four network clusters showing high similarity ($J > 0.02$) among edge compositions were identified (Fig. 5c). Cluster 1 consisted of interkingdom networks constructed from bulk soil and rhizosphere. Meanwhile, microbial networks identified in root endosphere and aboveground compartments belonged to Clusters 2, 3, and 4. Notably, microbial networks identified at the ripening stages clustered tightly with each other, whereas networks at the vegetative and reproductive stages did not show high similarity. On the other hand, seed microbial networks belonged to an isolated cluster (cluster 3). This cluster consisted of seed microbial networks identified at the sowing stage and ripening stages. Seed microbial networks at the booting stage (76 days after transplanting) and at the heading stage (90 days after transplanting) belonged to Cluster 2 and orphan group, respectively.

**Supplementary Result 3**

**Non-random distribution of microbial associations in plant compartments**

Co-occurrence patterns and non-random distribution patterns varied depending on rice compartments and soils (Supplementary Fig. 12). Associations governed by Proteobacteria, Firmicutes, Ascomycota, and Basidiomycota mostly consisted of the associations in leaf and stem endospheres (Supplementary Fig. 12). When the occurrence relationships of microbial associations were investigated at the higher taxonomic levels, associations among bacterial classes Gammaproteobacteria and Alphaproteobacteria (belonging to Proteobacteria) and fungal classes Dothideomycetes and Sordariomycetes compose microbial associations in the leaf endospheres (Supplementary Data 10). In the stem endospheres, stems collected at 60-70 cm (S7), 70-80cm (S8), and 80-90 cm (S9) showed similar occurrence relationship patterns with the leaf endospheres. Meanwhile, in the endosphere of S1 and S2 which were close to the soil, associations mediated by Bacteroidetes (class Bacteroidia) and Firmicutes (class Clostridia) were identified (Supplementary Fig. 12; Supplementary Data 10). Compared to aboveground compartments, associations with Acidobacteria (classes Subgroup 6 and Holophagae) and Chloroflexi (class Anaerolineae) were found. Using hypergeometric distribution analysis, associations with unidentified fungal classes were overrepresented than random frequency in rhizosphere and bulk soils. In the root endosphere, associations with the bacterial classes Deltaproteobacteria, Anaerolineae, and Bacilli were overrepresented (Supplementary Fig. 12; Supplementary Data 10). In seeds and leaves, there were no taxa showing the overrepresentation in intra-kingdom associations. Most of the inter-kingdom associations mediated by classes Bacilli, Actinobacteriia, Bacteroida, and Alphaproteobacteria showed non-random distribution (Supplementary Fig. 12). At the order level, associations between the fungal order Pleosporales and the bacterial orders Betaproteobacteriales, Sphingomonadales, and Xanthomonadales were commonly overrepresented than random frequency in seeds and leaves (Supplementary Data 10). The associations in S9 (stems collected at 80-90 cm) were randomly distributed at the order level (Supplementary Data 10). Meanwhile, in the belowground compartments (bulk soil, rhizosphere, and root), unidentified fungal order showed non-random distribution with other bacterial and fungal orders. Associations with Clostridiales, Anaerolineales, Bacteroidales, and Solibacterales further showed non-random distribution in the root endosphere (Supplementary Data 10).

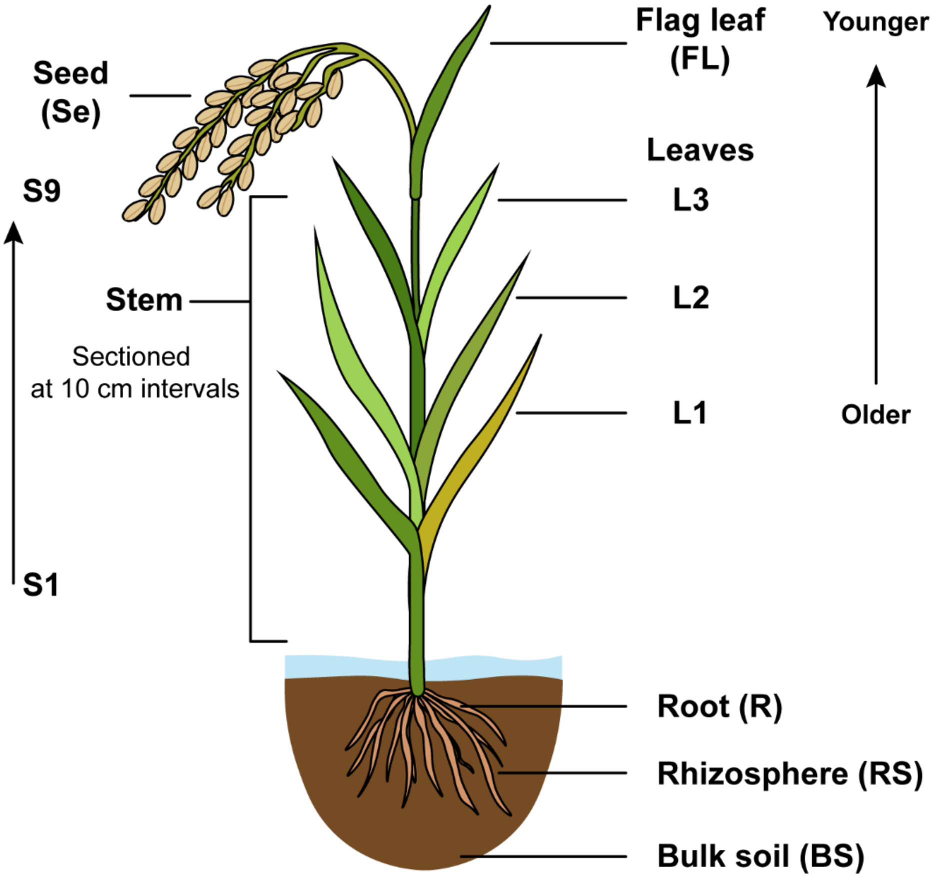


**Supplementary Fig. 1.**

**Sectioning of a rice plant in 2018 samples.** To track temporal changes in bacterial and fungal communities in plant compartments, rice plants were sectioned based on topological positions. In leaves, leaves located close to soils were designated as L1. And then, the sample names of leaves were assigned based on their positions. Leaves close to panicles were assigned to flag leaf (FL). S1 is the stem position close to soil (0-10 cm). And then, the names of other stem samples were designated at 10 cm intervals. Belowground compartments were also divided into three parts: root endosphere, rhizosphere, and bulk soils. Details on entire experiments and analyses were described in Materials and Methods.

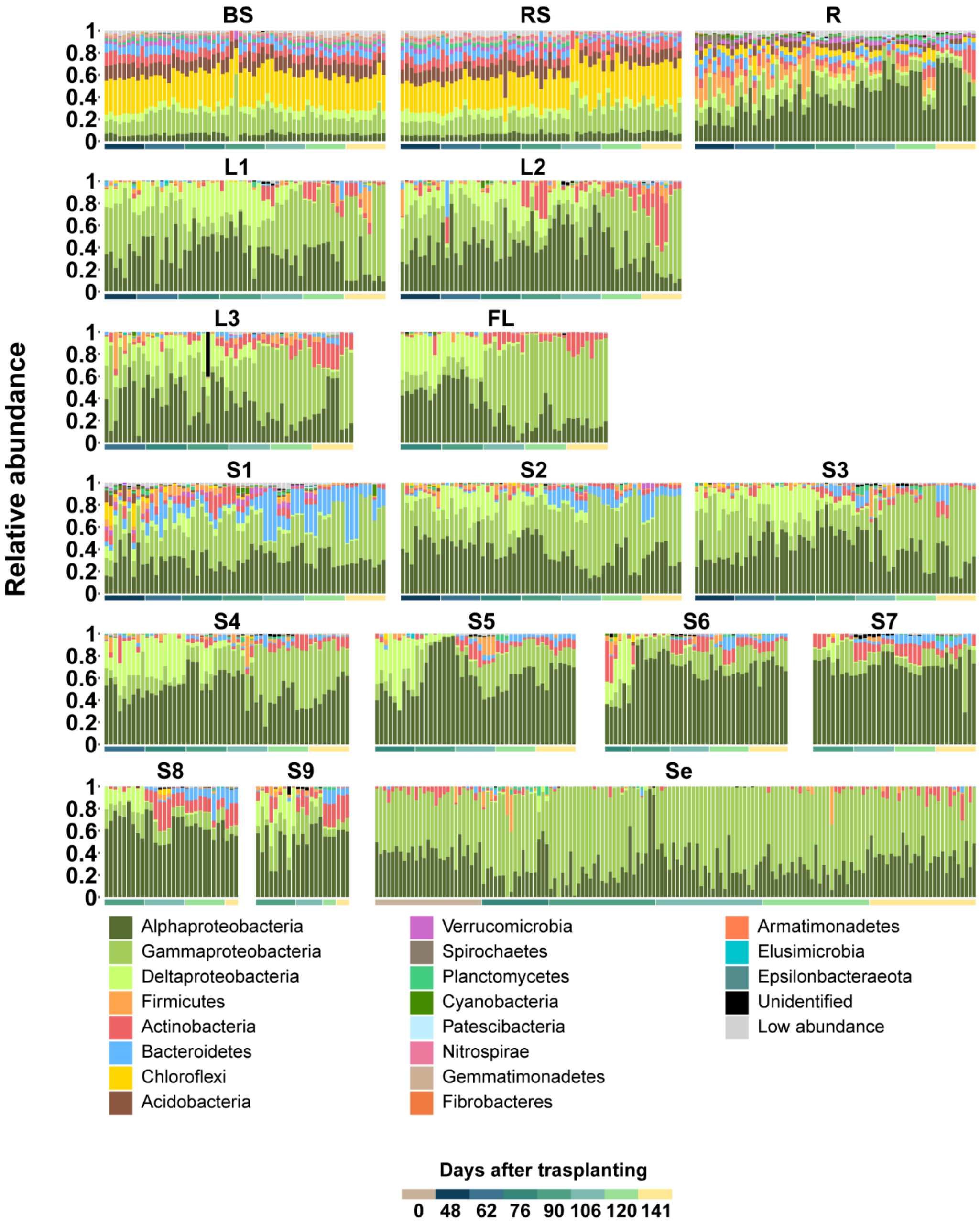


**Supplementary Fig. 2.**

**Composition of bacterial communities in each replicate collected in 2018.** Each bar represents relative abundance of each replicate of a particular age (days after transplanting). The bars are ordered by the sampling points as indicated by the colored bars under each bar. BS, bulk soil; RS, rhizosphere soil; R, root endosphere; FL, flag leaves which are located next to panicles; L3, the leaves located 10 cm below the flag leaves; L2, the leaves located 10 cm below the L3; L1, the leaves located 10 cm below the L2; S1-S9, stem samples which were also separated at 10 cm intervals depending on the height of plants; Se, seeds. Developmental stage: 0, sowing; 48, tillering; 62, tillering; 76, booting; 90, heading; 106, dough; 120, yellow-ripening; 141, harvest.

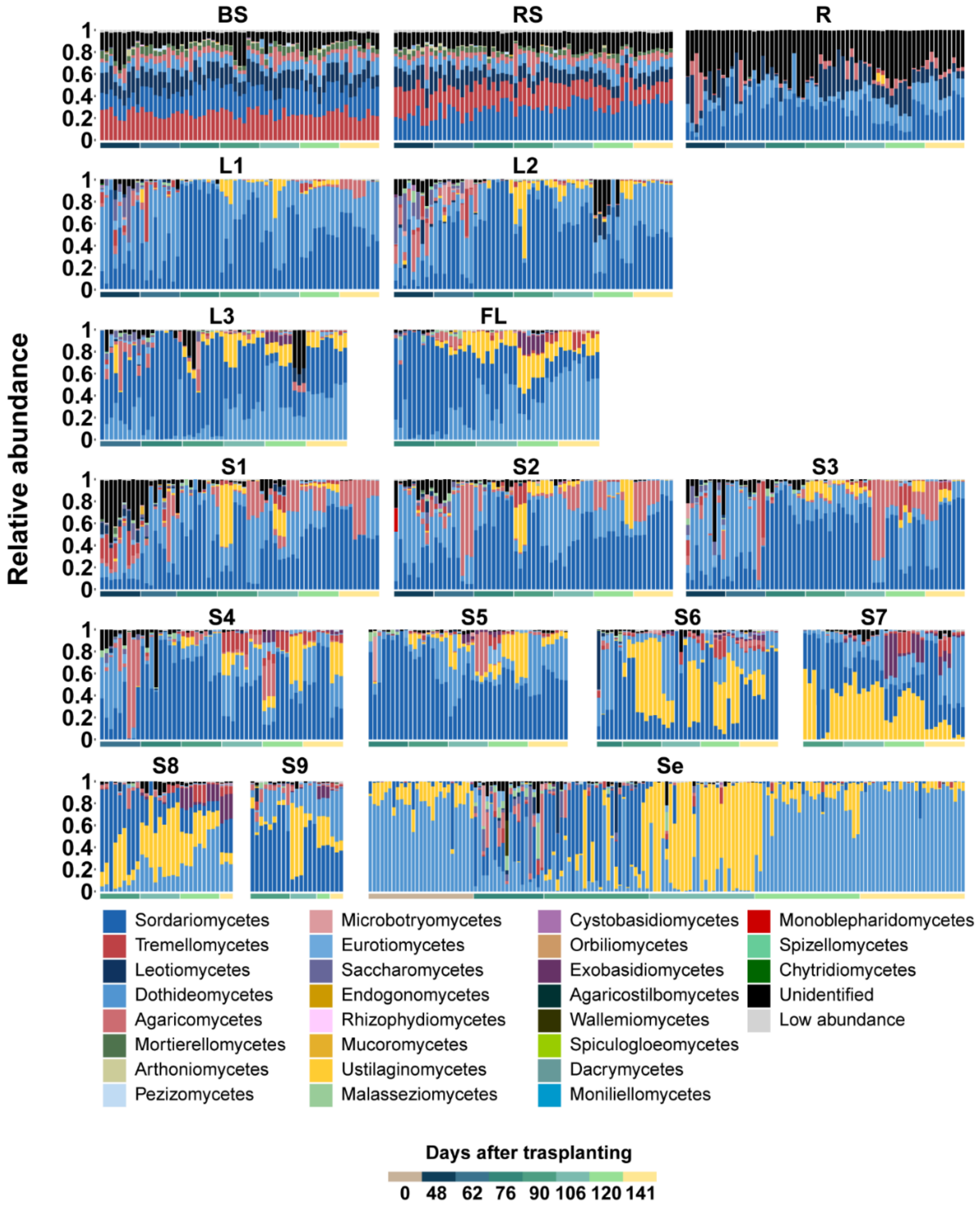


**Supplementary Fig. 3.**

**Composition of fungal communities in each replicate collected in 2018.** Each bar represents relative abundance of each replicate of a particular age (days after transplanting). The bars are ordered by the rice age as indicated by the colored bars under each bar. BS, bulk soil; RS, rhizosphere soil; R, root endosphere; FL, flag leaves which are located next to panicles; L3, the leaves located 10 cm below the flag leaves; L2, the leaves located 10 cm below the L3; L1, the leaves located 10 cm below the L2; S1-S9, stem samples which were also separated at 10 cm intervals depending on the height of plants; Se, seeds. Developmental stage: 0, sowing; 48, tillering; 62, tillering; 76, booting; 90, heading; 106, dough; 120, yellow-ripening; 141, harvest.

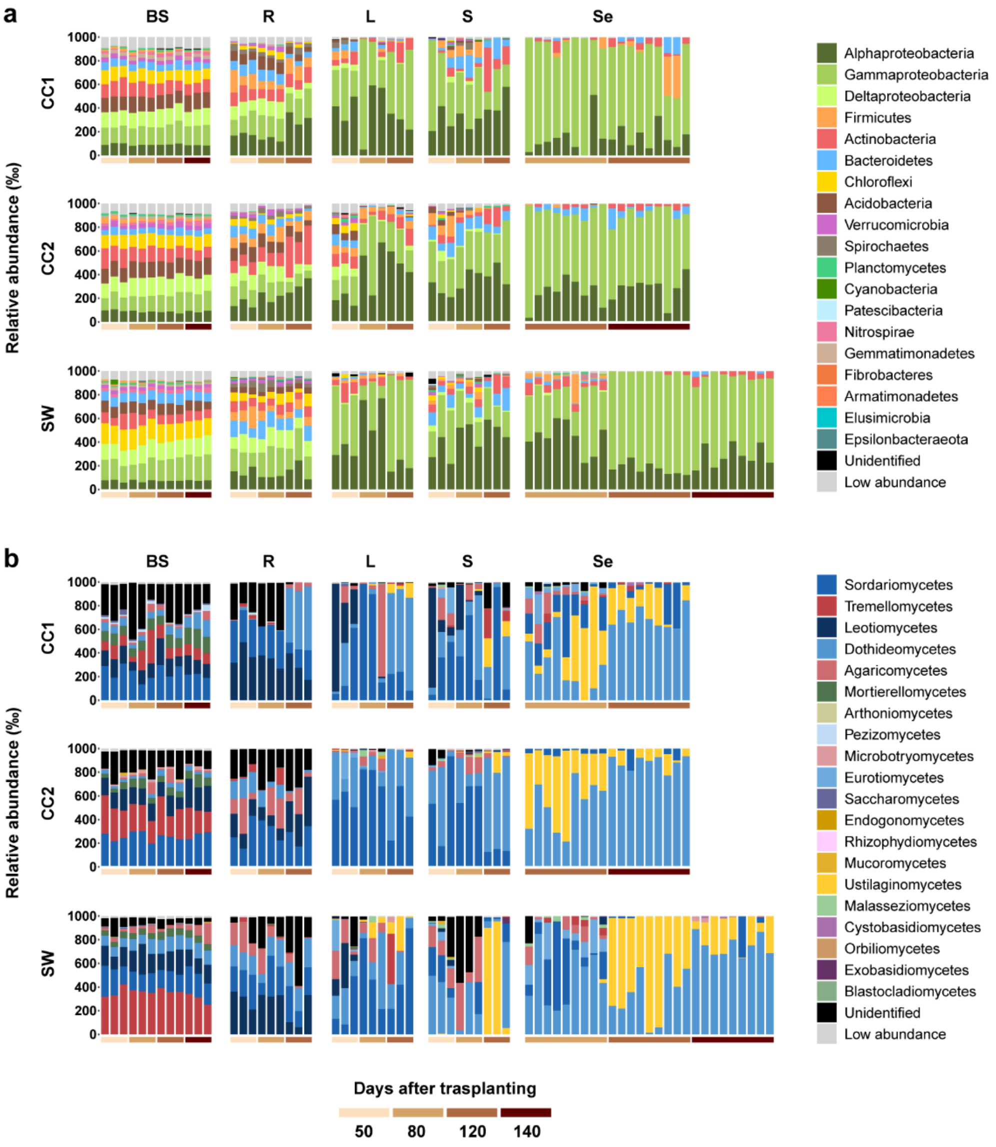


**Supplementary Fig. 4.**

**Relative abundances of rice-associated bacterial and fungal communities in the samples collected in 2017.** (**a**) Distribution of relative abundances of bacterial communities in different compartments during the growing season of rice in 2017. (**b**) Distribution of relative abundances of fungal communities in different compartments during the growing season of rice in 2017. The rice-associated microbial communities were investigated in three different soil sites (SW, Suwon; CC1, Chuncheon 1; CC2, Chuncheon 2). Each bar represents 1 sample of a particular age (days after transplanting). The bars are ordered by the rice age as indicated by the colored bars under each bar. BS, bulk soil; R, root endosphere; L, leaf endosphere; S, stem endosphere; Se, seeds. Developmental stage: 50, tillering; 80, heading; 120, harvest for CC1 field (cultivar Gohyanchal, faster-growing rice); 50, tillering; 80, booting; 120, yellow-ripening; 140, harvest for CC2 field (cultivar Daean, slower-growing rice); 50, tillering; 80, heading; 120, yellow-ripening; 140, harvest for SW field (cultivar Akibare, slower-growing rice).

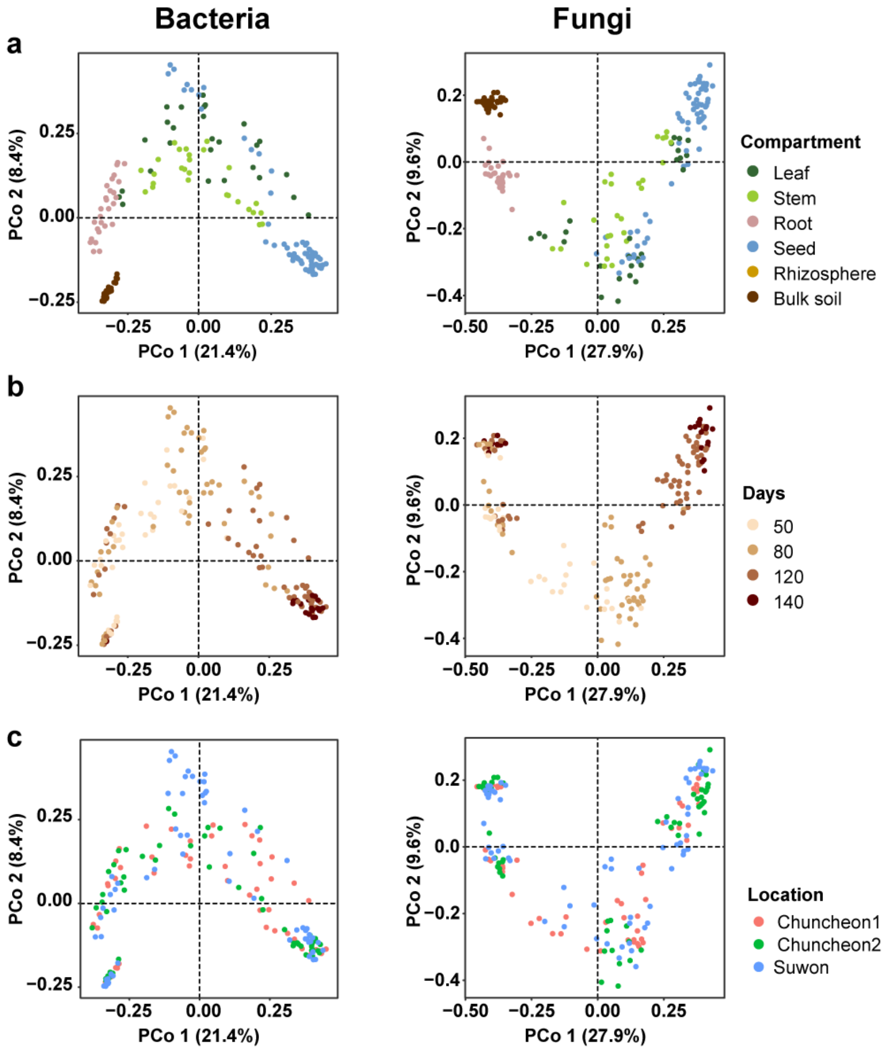


**Supplementary Fig. 5.**

**Principal coordinate analysis of bacterial and fungal communities in rice samples collected in 2017.** Compositional variations among samples were estimated via principal coordinate analysis (PCoA) based on Bray-Curtis distance metric. (**a**) The samples are colored by compartments (leaf, stem, root, seed, rhizosphere, and bulk soil). (**b**) The rice samples are grouped according to age. (**c**) The rice samples are divided by geographic locations. Developmental stage: 50, tillering; 80, heading; 120, harvest for CC1 field (cultivar Gohyanchal, faster-growing rice); 50, tillering; 80, booting; 120, yellow-ripening; 140, harvest for CC2 field (cultivar Daean, slower-growing rice); 50, tillering; 80, heading; 120, yellow-ripening; 140, harvest for SW field (cultivar Akibare, slower-growing rice).

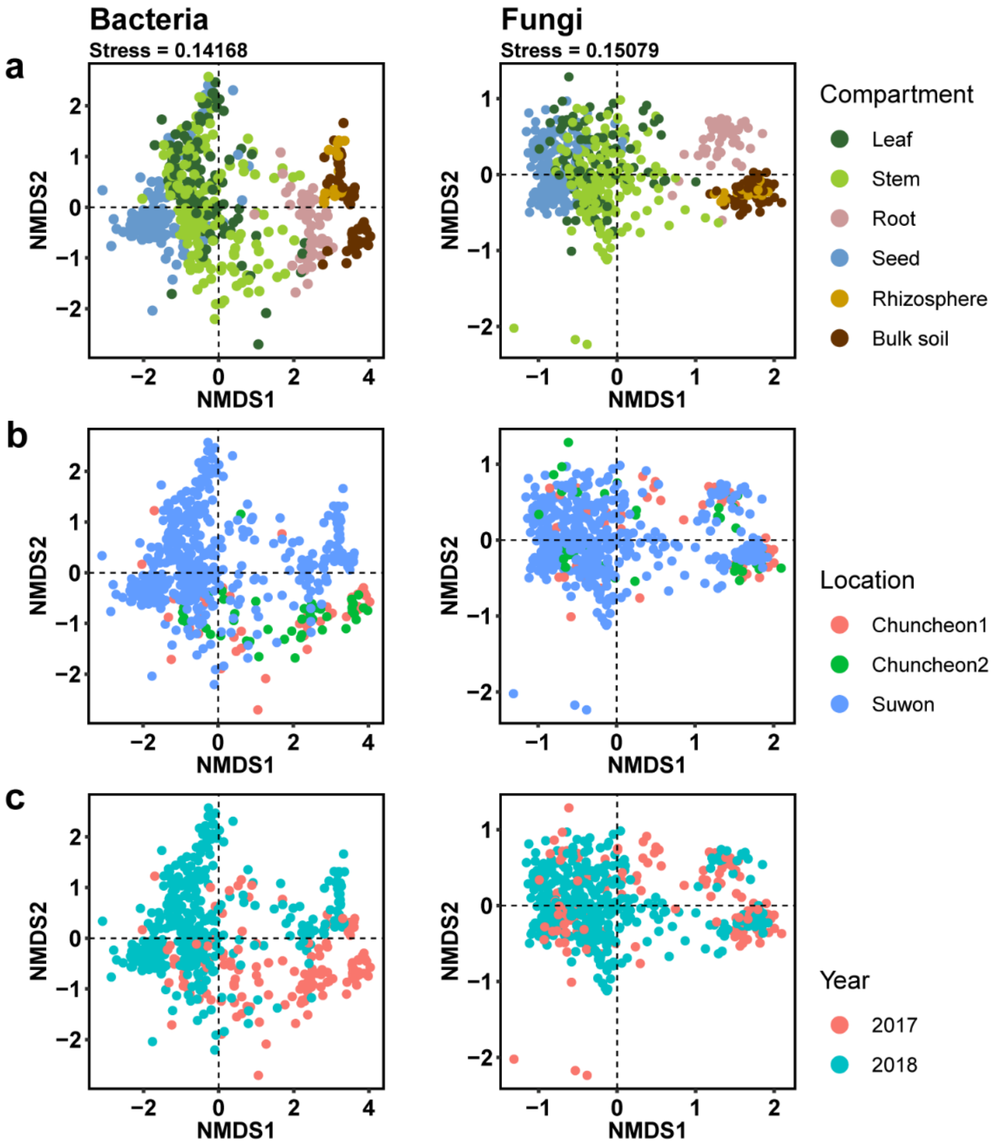


**Supplementary Fig. 6.**

**Non-metric multidimensional scaling analysis of bacterial and fungal communities in rice samples collected in 2017 and 2018.** Compositional variations among samples were estimated via non-metric multidimensional scaling (NMDS) analysis based on Bray-Curtis distance metric. Bray-Curtis distance was calculated from the mean abundance tables of technical replicates. (**a**) The samples are colored by compartments (leaf, stem, root, seed, rhizosphere, and bulk soil). (**b**) The rice samples are grouped according to location (soil sites). (**c**) The rice samples are divided by the sampling year. Dots indicate biological replicates of each plant compartment-associated bacterial or fungal communities.

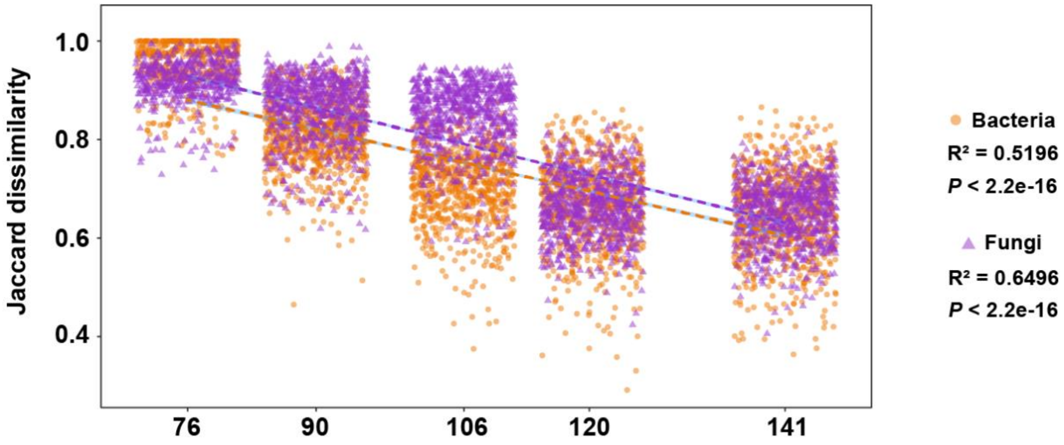


**Supplementary Fig. 7.**

**Community dissimilarity between parent seeds and other developing progeny seeds.** Pairwise community dissimilarity between parent seeds (Se0, seeds at the sowing stage) and progeny seeds is calculated by Jaccard distance. *x*-axis indicates days after transplanting. The dissimilarity values of bacterial and fungal communities are indicated as orange circles and purple triangles, respectively. Trend lines of temporal changes in dissimilarity values are estimated using a linear regression analysis. An orange and purple dashed lines represent bacterial and fungal communities, respectively.

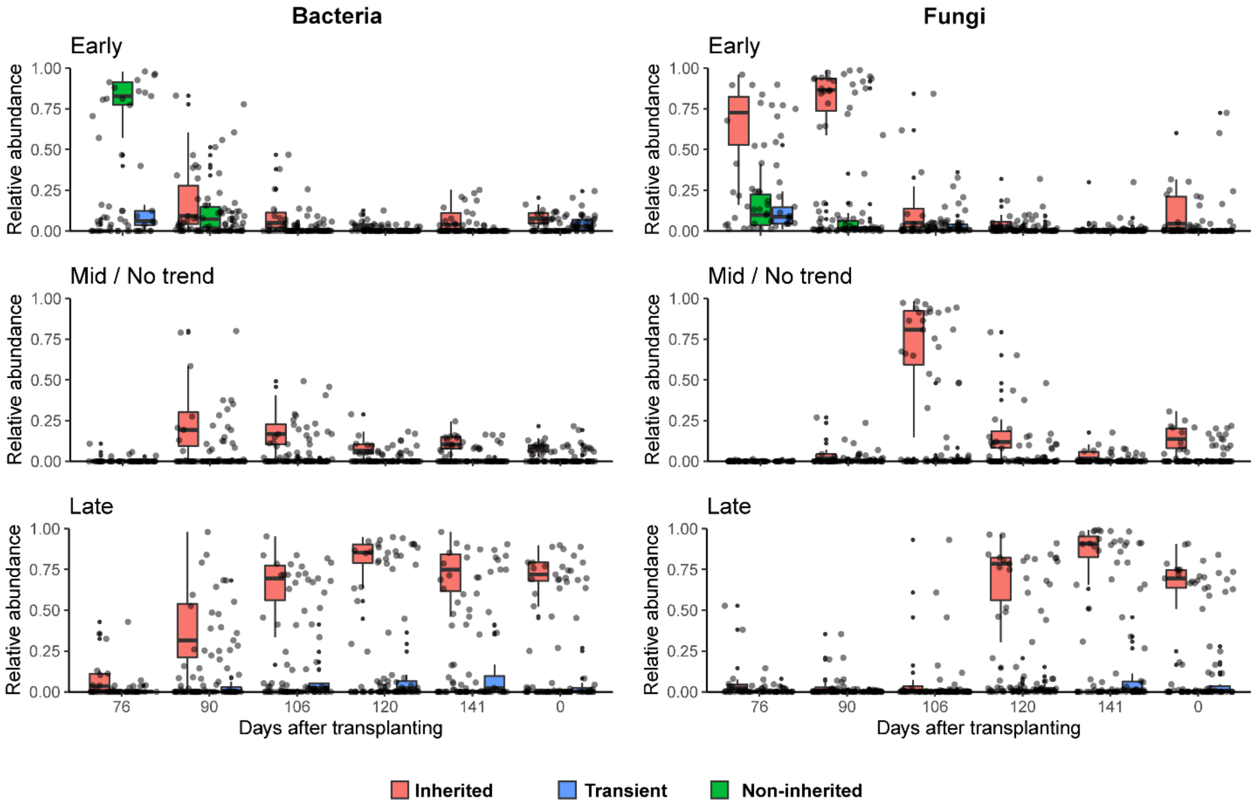


**Supplementary Fig. 8.**

**Successional modes of seed bacterial and fungal communities.** Succession modes were classified based on linear regression between time and relative abundance of each OTU (Early, t < 0, *P* < 0.05; Mid/no trend, *P* > 0.05; Late, t > 0, *P* < 0.05). The numbers in x-axis indicate sampling point (days after transplanting to fields). The number 0 indicates parent seeds. The other numbers indicate progeny seeds. Colors of boxes indicate three groups of seed microbial community (magenta, vertically transmitted OTU; blue, transient OTU; green, non-transmitted OTU). Boxes and lines in the boxes represent inter-quantile range (Q3-Q1) and median of the cumulative relative abundances of vertically transmitted, transient, and non-inherited OTUs, respectively. Black-filled and grey dots indicate potential outliers and the cumulative abundances of each inheritance group in each biological replicate, respectively. Lower and upper whiskers show minimum and maximum values of cumulative relative abundance in each group.

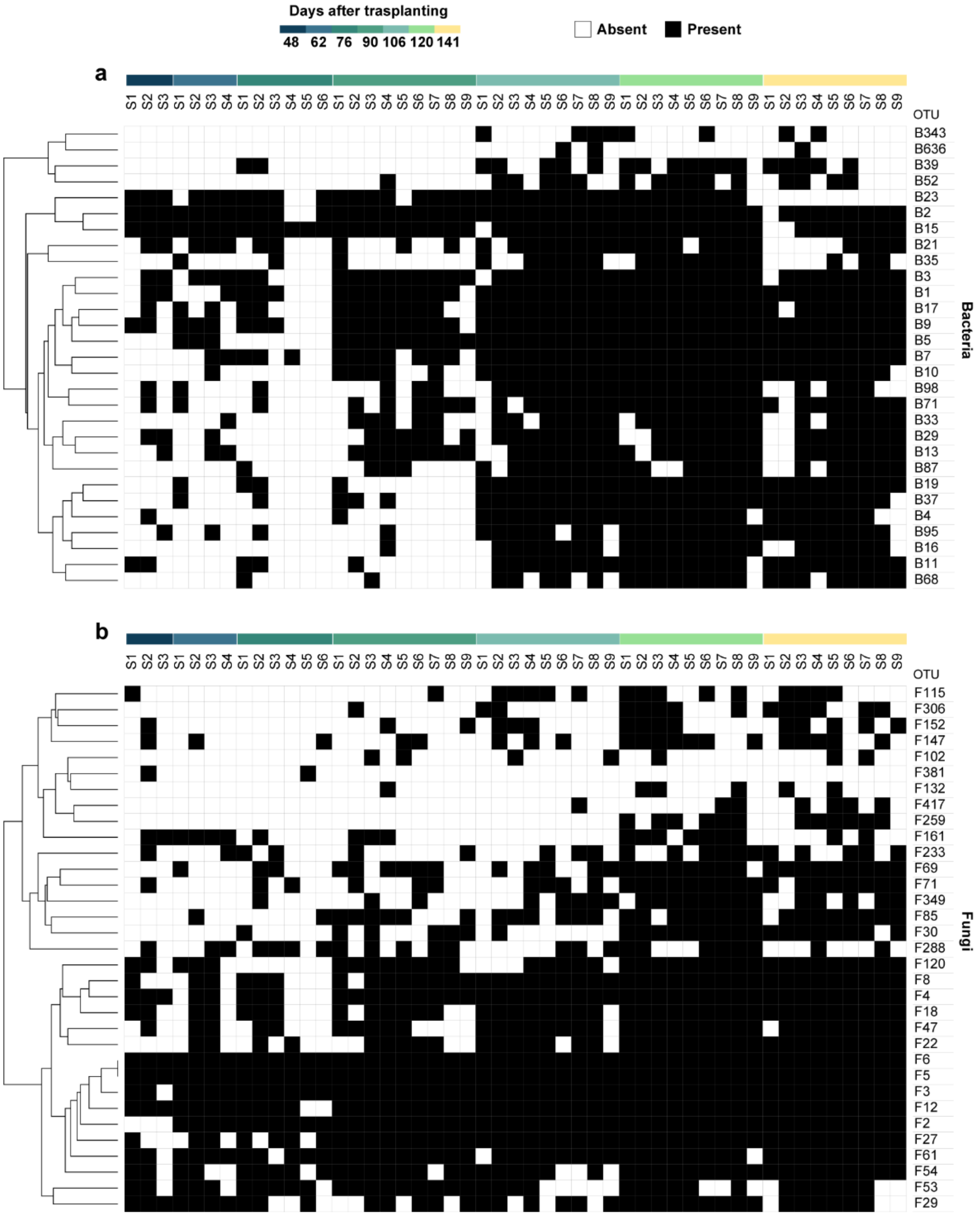


**Supplementary Fig. 9.**

**Presence and absence patterns of vertically transmitted bacterial and fungal communities in stem sections.** (**a**) Presence and absence patterns of vertically transmitted bacterial community. (**b**) Presence and absence patterns of vertically transmitted fungal community. Heat maps were constructed using Morpheus (https://software.broadinstitute.org/morpheus/). Colored bars above heat maps correspond to the sampling timepoints when stem sections were collected. White and black boxes indicate absence and presence of OTUs in each stem section in a particular sampling time, respectively. The pattern profiles were clustered by hierarchical clustering based on Jaccard distance implemented in Morpheus. Developmental stage: 48, tillering; 62, tillering; 76, booting; 90, heading; 106, dough; 120, yellow-ripening; 141, harvest.

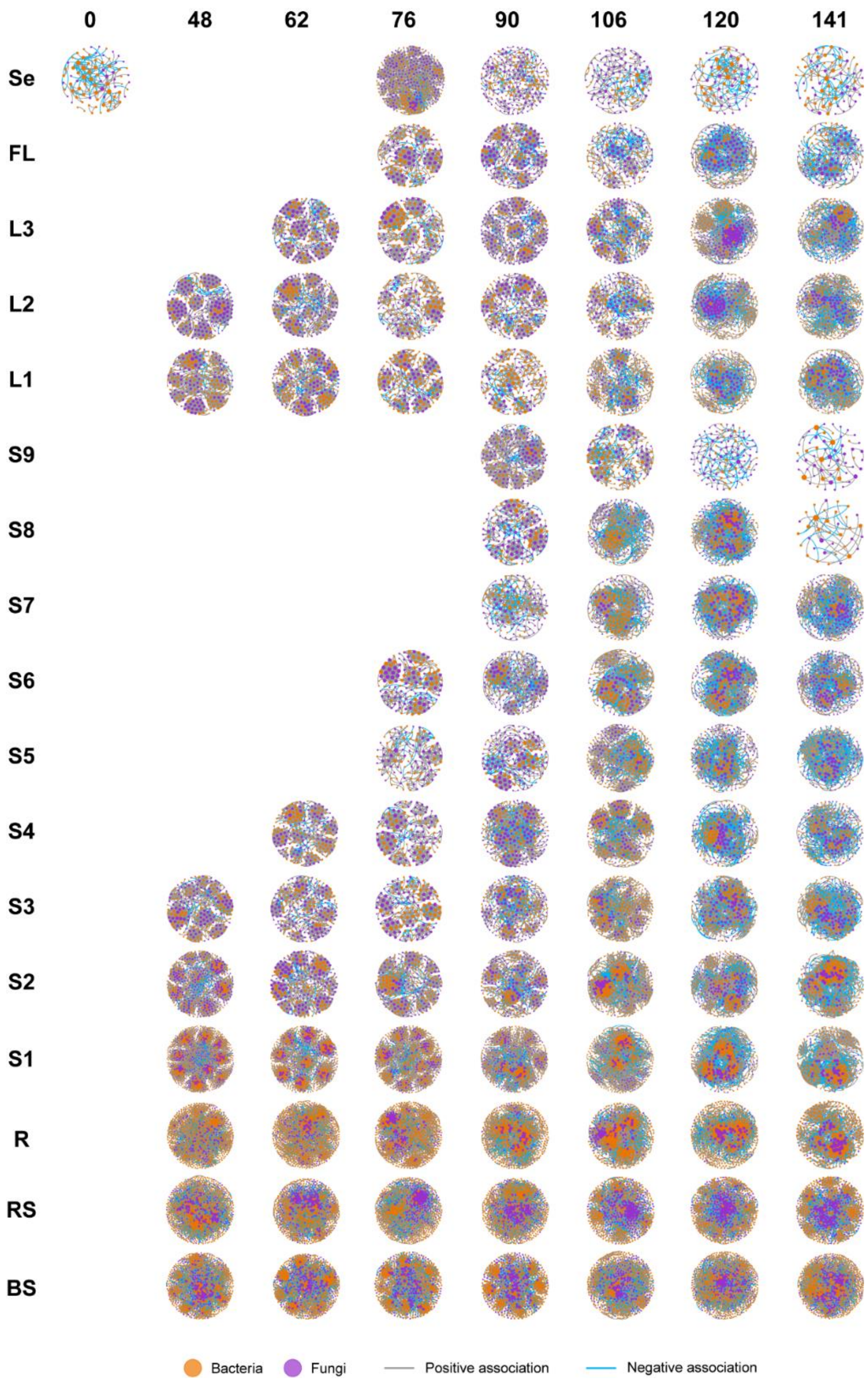


**Supplementary Fig. 10.**

**Spatiotemporal dynamics of inter-kingdom networks of bacterial and fungal communities.** Significant correlations showing $P < 0.05$ with correlation coefficient > 0.3 or < -0.3 were selected. Positive and negative associations are colored by grey and light blue, respectively. The size of circles is proportional to the degree centrality of each node. Orange circles indicate bacterial nodes, whereas purple circles represent fungal nodes. The numbers indicate days after transplanting. Se, seeds; FL, flag leaves which are located next to panicles; L3, the leaves located

at 30-40 cm from soil; L2, the leaves located at 20-30 cm from soil; L1, the leaves located at 10-20 cm from soil; S1-S9, stem samples which were also separated at 10 cm intervals depending on the height of plants. S1, stem endosphere located at 0-10 cm from soil; S2, stem endosphere located at 10-20 cm from soil; S3, stem endosphere located at 20-30 cm from soil; S4, stem endosphere located at 30-40 cm from soil ; S5, stem endosphere located at 40-50 cm from soil ; S6, stem endosphere located at 50-60 cm from soil ; S7, stem endosphere located at 60-70 cm from soil ; S8, stem endosphere located at 70-80 cm from soil; S9, stem endosphere located above 80 cm from soil; BS, bulk soil; RS, rhizosphere soil; R, root endosphere. Developmental stage: 0, sowing; 48, tillering; 62, tillering; 76, booting; 90, heading; 106, dough; 120, yellow-ripening; 141, harvest.

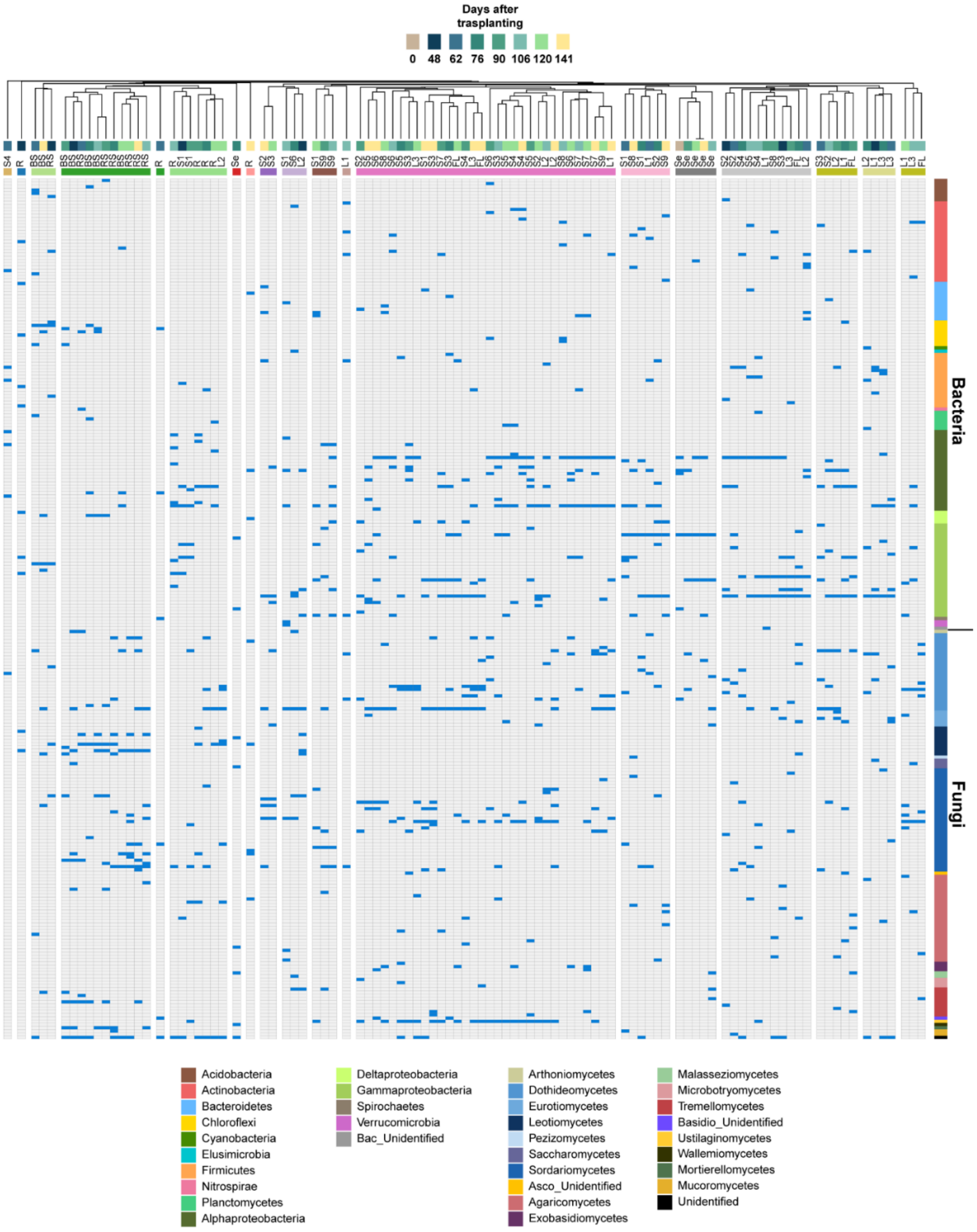


**Supplementary Fig. 11.**

**Hierarchical clustering of hub nodes identified in bacterial-fungal cooccurrence networks.** Network hubs are simply defined as nodes that ranked in top 10 based on degree centrality. The presence of hubs is indicated as blue boxes in the heat map. Samples were clustered based on the presence and absence of hubs using Jaccard distance. Colored boxes right under the tips of the dendrogram denote host age (days after transplanting). Colored bars under the name of rice

compartments indicate the clusters. Taxonomic affiliation of hubs is depicted as the colored bars right on the heat map. Se, seeds; FL, flag leaves which are located next to panicles; L3, the leaves located at 30-40 cm from soil; L2, the leaves located at 20-30 cm from soil; L1, the leaves located at 10-20 cm from soil; S1-S9, stem samples which were also separated at 10 cm intervals depending on the height of plants. S1, stem endosphere located at 0-10 cm from soil; S2, stem endosphere located at 10-20 cm from soil; S3, stem endosphere located at 20-30 cm from soil; S4, stem endosphere located at 30-40 cm from soil ; S5, stem endosphere located at 40-50 cm from soil ; S6, stem endosphere located at 50-60 cm from soil ; S7, stem endosphere located at 60-70 cm from soil ; S8, stem endosphere located at 70-80 cm from soil ; S9, stem endosphere located above 80 cm from soil; BS, bulk soil; RS, rhizosphere soil; R, root endosphere. Developmental stage: 0, sowing; 48, tillering; 62, tillering; 76, booting; 90, heading; 106, dough; 120, yellow-ripening; 141, harvest.

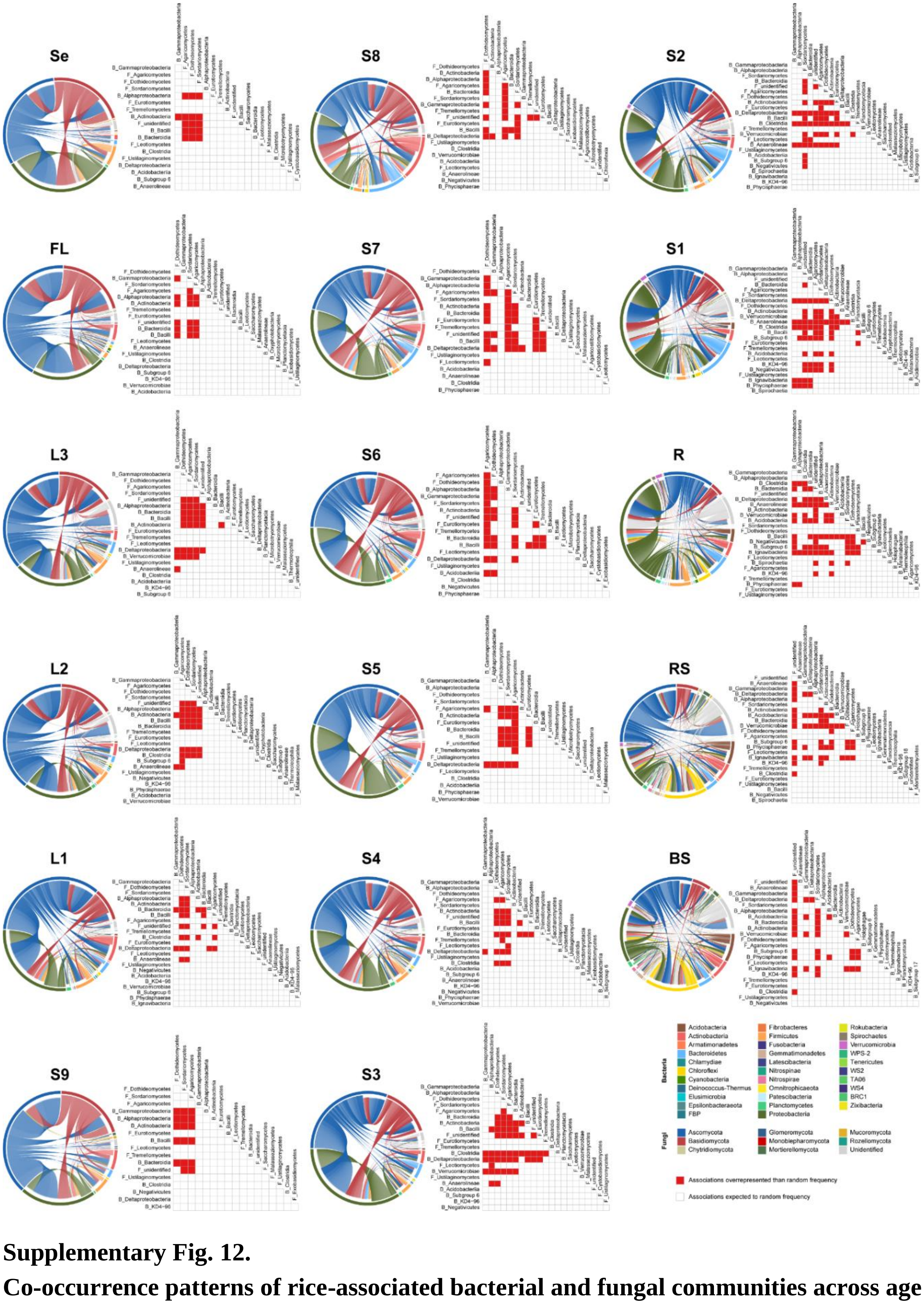


**Supplementary Fig. 12.**

**Co-occurrence patterns of rice-associated bacterial and fungal communities across age and compartments.** The taxonomic profiles of bacterial-fungal co-occurrence associations (edges) of

dominant phyla and classes. Interconnections of bacterial and fungal taxa are indicated as edges in a directed network. Edges are lines that connect “Source node” taxa with which edges start and “Target node” taxa with which edges end. Edges are colored by the taxon acting as a source node. Non-random distribution of bacterial-fungal associations across age and compartments at the class level. Overrepresentation of microbial associations was estimated using the hypergeometric distribution analysis. Kingdoms where each class belongs are indicated as “B_” for bacteria and “F_” for fungi. Overrepresented associations are colored by red squares, whereas randomly distributed associations are colored by white squares. Se, seeds; FL, flag leaves which are located next to panicles; L3, the leaves located at 30-40 cm from soil; L2, the leaves located at 20-30 cm from soil; L1, the leaves located at 10-20 cm from soil; S1-S9, stem samples which were also separated at 10 cm intervals depending on the height of plants. S1, stem endosphere located at 0-10 cm from soil; S2, stem endosphere located at 10-20 cm from soil; S3, stem endosphere located at 20-30 cm from soil; S4, stem endosphere located at 30-40 cm from soil ; S5, stem endosphere located at 40-50 cm from soil ; S6, stem endosphere located at 50-60 cm from soil ; S7, stem endosphere located at 60-70 cm from soil ; S8, stem endosphere located at 70-80 cm from soil ; S9, stem endosphere located above 80 cm from soil; BS, bulk soil; RS, rhizosphere soil; R, root endosphere.

**Supplementary Table 1.**
**Primers used in this study**

| | Primer | Sequence |
|---|---|---|
| **Bacteria** | 515F | 5'-TCGTCGGCAGCGTCAGATGTGTATAAGAGACAGGTGCCAGCMGCCGCGGTAA-3' |
| | 806R | 5'-GTCTCGTGGGCTCGGAGATGTGTATAAGAGACAGGGACTACHVGGGTWTCTAAT-3' |
| **Fungi** | ITS3 | 5'-TCGTCGGCAGCGTCAGATGTGTATAAGAGACAGGCATCGATGAAGAACGCAGC-3' |
| | ITS4 | 5'-GTCTCGTGGGCTCGGAGATGTGTATAAGAGACAGTCCTCCGCTTATTGATATGC-3' |

**Supplementary Table 2.**
**PCR blockers used in this study**

| Name | Sequence |
| --- | --- |
| **"mPNA" Mitochondrial Blocker** | 5'-GGCAAGTGTTCTTCGGA-3' |
| **"pPNA" Plastid Blocker** | 5'-GGCTCAACCCTGGACAG-3' |
