# Supplementary material for: Longitudinal transmission of bacterial and fungal communities from seed to seed in rice
Source: Commun Biol. 2022 Aug 1;5:772. doi: 10.1038/s42003-022-03726-w (PMC9343636; doi:10.1038/s42003-022-03726-w)
Supplement: Supplementary file 3 — Description of Additional Supplementary Data [file 42003_2022_3726_MOESM3_ESM.pdf]

Description of Additional Supplementary Files

File name: Supplementary Data 1
Description: Community structure and related factors on bacterial and fungal communities.

File name: Supplementary Data 2
Description: Taxonomy of vertically transmitted bacterial and fungal OTUs.

File name: Supplementary Data 3
Description: Succession mode of seed microbial OTUs predicted using a linear regression analysis.

File name: Supplementary Data 4
Description: Results on the test of Sloan's neutral model.

File name: Supplementary Data 5
Description: List of core bacterial and fungal OTUs in bulk soil, rhizosphere, root, stem, leaf, and seed.

File name: Supplementary Data 6
Description: Numbers of nodes and edges of microbial networks of rice compartments.

File name: Supplementary Data 7
Description: Composition of edges consisting of top 4 modules in the meta-network.

File name: Supplementary Data 8
Description: Profile of top 10 degree in 101 bacterial-fungal co-occurrence networks.

File name: Supplementary Data 9
Description: Co-occurrence relationships and non-random distribution of bacterial-fungal associations at the metacommunity network level.

File name: Supplementary Data 10
Description: Co-occurrence relationships and non-random distribution of bacterial and fungal associations in each rice compartment.

File name: Supplementary Data 11
Description: Metadata of bacterial and fungal communities.
