# Supplementary material for: Longitudinal transmission of bacterial and fungal communities from seed to seed in rice
Source: Commun Biol. 2022 Aug 1;5:772. doi: 10.1038/s42003-022-03726-w (PMC9343636; doi:10.1038/s42003-022-03726-w)
Supplement: Supplementary file 15 — Reporting Summary [file 42003_2022_3726_MOESM15_ESM.pdf]

nature portfolio

Corresponding author(s): Yong-Hwan Lee

Last updated by author(s): Jun 22, 2022

Reporting Summary

Nature Portfolio wishes to improve the reproducibility of the work that we publish. This form provides structure for consistency and transparency in reporting. For further information on Nature Portfolio policies, see our Editorial Policies and the Editorial Policy Checklist.

Statistics

For all statistical analyses, confirm that the following items are present in the figure legend, table legend, main text, or Methods section.

n/a	Confirmed	
☐	☒	The exact sample size (*n*) for each experimental group/condition, given as a discrete number and unit of measurement
☐	☒	A statement on whether measurements were taken from distinct samples or whether the same sample was measured repeatedly
☐	☒	The statistical test(s) used AND whether they are one- or two-sided<br>*Only common tests should be described solely by name; describe more complex techniques in the Methods section.*
☒	☐	A description of all covariates tested
☐	☒	A description of any assumptions or corrections, such as tests of normality and adjustment for multiple comparisons
☐	☒	A full description of the statistical parameters including central tendency (e.g. means) or other basic estimates (e.g. regression coefficient) AND variation (e.g. standard deviation) or associated estimates of uncertainty (e.g. confidence intervals)
☐	☒	For null hypothesis testing, the test statistic (e.g. *F*, *t*, *r*) with confidence intervals, effect sizes, degrees of freedom and *P* value noted<br>*Give P values as exact values whenever suitable.*
☒	☐	For Bayesian analysis, information on the choice of priors and Markov chain Monte Carlo settings
☒	☐	For hierarchical and complex designs, identification of the appropriate level for tests and full reporting of outcomes
☒	☐	Estimates of effect sizes (e.g. Cohen's *d*, Pearson's *r*), indicating how they were calculated

Our web collection on statistics for biologists contains articles on many of the points above.

Software and code

Policy information about availability of computer code

Data collection	No software was used to collect data in this study
Data analysis	QIIME 2: https://qiime2.org/<br>DADA2: https://benjjneb.github.io/dada2/index.html<br>vsearch: https://github.com/qiime2/q2-vsearch<br>R: https://www.r-project.org/<br>R, metagenomeSeq package: https://www.bioconductor.org/packages/release/bioc/html/metagenomeSeq.html<br>R, microbiome package: https://bioconductor.org/packages/devel/bioc/vignettes/microbiome/inst/doc/vignette.html<br>R, vegan package: https://cran.r-project.org/web/packages/vegan/vegan.pdf<br>R, igraph package: https://cran.r-project.org/web/packages/igraph/igraph.pdf<br>R, lme4 package: https://cran.r-project.org/web/packages/lme4/lme4.pdf<br>R, brainGraph: https://cran.r-project.org/web/packages/brainGraph/brainGraph.pdf<br>FastSpar: https://github.com/scwatts/fastspar<br>Code & raw data: https://github.com/hyunkim90/spatiotemporal_tracking_rice_endophytic_communities

For manuscripts utilizing custom algorithms or software that are central to the research but not yet described in published literature, software must be made available to editors and reviewers. We strongly encourage code deposition in a community repository (e.g. GitHub). See the Nature Portfolio guidelines for submitting code & software for further information.

Data

Policy information about availability of data

All manuscripts must include a data availability statement. This statement should provide the following information, where applicable:

- Accession codes, unique identifiers, or web links for publicly available datasets
- A description of any restrictions on data availability
- For clinical datasets or third party data, please ensure that the statement adheres to our policy

Sequencing data at NCBI: BioProject ID PRJNA728671, PRJNA728647, and PRJNA733292

Field-specific reporting

Please select the one below that is the best fit for your research. If you are not sure, read the appropriate sections before making your selection.

☐ Life sciences ☐ Behavioural & social sciences ☒ Ecological, evolutionary & environmental sciences

For a reference copy of the document with all sections, see nature.com/documents/nr-reporting-summary-flat.pdf

Ecological, evolutionary & environmental sciences study design

All studies must disclose on these points even when the disclosure is negative.

Study description	Spatial and temporal monitoring of bacterial and fungal communities associated with rice plants grown under field conditions across two successive years. We examined inheritance of bacterial and fungal communities during rice development. We also investigated ecological forces governing the microbial inheritance. In addition, using a metacommunity network, temporal community dynamics was explored.
Research sample	Soils and rice compartment samples were collected from 3 rice paddy fields at 2 geographically distant sites in 2017 and 2018. In 2017, a total of 180 samples (36 soil samples, 27 leaf samples, 27 stem samples, 27 root samples, and 63 seed samples) were collected once a month during rice development. In 2018, a total of 984 soil and rice compartment samples (63 bulk soil samples, 63 rhizosphere samples, 63 root endosphere samples, 225 leaf samples, 417 stem samples, and 153 seed samples) were collected (n = 1,146 samples in total).
Sampling strategy	To avoid the effects of plant debris, soils below a depth of 5 cm were removed. Two kg of soils below a depth of 15 cm were collected from each field. The collected soils were sieved through a 2 mm mesh to remove plant debris and particles larger than sand grains. For collecting rhizosphere and root samples, we adopted the collection scheme from the work of Edwards et al., 2015 (PNAS). The divided leaf and stem samples were surface-sterilized. In 2018, leaf and stem samples were further divided based on the position and height. Details for sampling strategy are described in the Methods section.
Data collection	Isolation of DNAs from soil samples, as well as library preparation are described in the Methods section. Bacterial and fungal communities were profiled using primer pairs targeting the V4 regions of the bacterial and archaeal 16S rRNA gene and the ITS2 segments of the fungal ITS. To inhibit the amplification of mitochondrial and plastid DNAs of rice, pPNA and mPNA were added in PCR mixtures. A total of 2,328 bacterial and fungal community profiles were generated. Sequencing was performed at National Instrumentation Center for Environmental Management (NICEM) at Seoul National University, Korea.
Timing and spatial scale	Soils and rice samples were collected in three fields in two geographic sites (94.4 km away). In 2017, soil and plant samples were collected in 50 (July), 80 (August), 120 (September), and 140 (October) days after transplanting. In 2018, samples were collected in 48, 62, 76, 90, 106, 120, and 141 days after transplanting.
Data exclusions	Very few samples where 16S rRNA or ITS sequences did not meet predetermined quality checks (i.e. low quality or low read counts) were excluded.
Reproducibility	All attempts to repeat the same sampling strategies across years were successful.
Randomization	Soils and rice samples were randomly obtained across sites to maximize the representation of the samples taken.
Blinding	Blinding was not relevant for this study as soils were collected regardless of their state.

Did the study involve field work? ☒ Yes ☐ No

Field work, collection and transport

Field conditions	Information on temperature, humidity, and soil conditions (flooded or dried) of fields is available in the Supplementary Data 11.
Location	GPS coordinates and information of sites can be found in the Methods section.
Access & import/export	Collaborators of each sampling site were contacted in order to identify rice paddy fields and access them. Collected samples were

Access & import/export	then transported to the laboratory in ice.
Disturbance	Soil was disturbed when soil samples were collected at each site. In order to minimize disturbance, holes made were covered with surrounding soil.

Reporting for specific materials, systems and methods

We require information from authors about some types of materials, experimental systems and methods used in many studies. Here, indicate whether each material, system or method listed is relevant to your study. If you are not sure if a list item applies to your research, read the appropriate section before selecting a response.

Materials & experimental systems

n/a	Involved in the study
☒	☐ Antibodies
☒	☐ Eukaryotic cell lines
☒	☐ Palaeontology and archaeology
☒	☐ Animals and other organisms
☒	☐ Human research participants
☒	☐ Clinical data
☒	☐ Dual use research of concern

Methods

n/a	Involved in the study
☒	☐ ChIP-seq
☒	☐ Flow cytometry
☒	☐ MRI-based neuroimaging
